# A hybrid sensory feedback system for thermal nociceptive warning and protection in prosthetic hand

**DOI:** 10.3389/fnins.2024.1351348

**Published:** 2024-04-08

**Authors:** Anran Xie, Chen Li, Chih-hong Chou, Tie Li, Chenyun Dai, Ning Lan

**Affiliations:** ^1^Laboratory of NeuroRehabilitation Engineering, School of Biomedical Engineering, Shanghai Jiao Tong University, Shanghai, China; ^2^Institute of Medical Robotics, School of Biomedical Engineering Shanghai Jiao Tong University, Shanghai, China; ^3^i-Lab, Suzhou Institute of Nano-Tech and Nano-Bionics (SINANO), Chinese Academy of Sciences (CAS), Suzhou, China; ^4^School of Biomedical Engineering, Shanghai Jiao Tong University, Shanghai, China; ^5^Richard and Loan Hill Department of Biomedical Engineering, University of Illinois Chicago, Chicago, IL, United States

**Keywords:** prosthetic hand, thermal protection, nociceptive perception, withdrawal reflex, transcutaneous electrical nerve stimulation

## Abstract

**Background:**

Advanced prosthetic hands may embed nanosensors and microelectronics in their cosmetic skin. Heat influx may cause damage to these delicate structures. Protecting the integrity of the prosthetic hand becomes critical and necessary to ensure sustainable function. This study aims to mimic the sensorimotor control strategy of the human hand in perceiving nociceptive stimuli and triggering self-protective mechanisms and to investigate how similar neuromorphic mechanisms implemented in prosthetic hand can allow amputees to both volitionally release a hot object upon a nociceptive warning and achieve reinforced release via a bionic withdrawal reflex.

**Methods:**

A steady-state temperature prediction algorithm was proposed to shorten the long response time of a thermosensitive temperature sensor. A hybrid sensory strategy for transmitting force and a nociceptive temperature warning using transcutaneous electrical nerve stimulation based on evoked tactile sensations was designed to reconstruct the nociceptive sensory loop for amputees. A bionic withdrawal reflex using neuromorphic muscle control technology was used so that the prosthetic hand reflexively opened when a harmful temperature was detected. Four able-bodied subjects and two forearm amputees randomly grasped a tube at the different temperatures based on these strategies.

**Results:**

The average prediction error of temperature prediction algorithm was 8.30 ± 6.00%. The average success rate of six subjects in perceiving force and nociceptive temperature warnings was 86.90 and 94.30%, respectively. Under the reinforcement control mode in Test 2, the median reaction time of all subjects was 1.39 s, which was significantly faster than the median reaction time of 1.93 s in Test 1, in which two able-bodied subjects and two amputees participated. Results demonstrated the effectiveness of the integration of nociceptive sensory strategy and withdrawal reflex control strategy in a closed loop and also showed that amputees restored the warning of nociceptive sensation while also being able to withdraw from thermal danger through both voluntary and reflexive protection.

**Conclusion:**

This study demonstrated that it is feasible to restore the sensorimotor ability of amputees to warn and react against thermal nociceptive stimuli. Results further showed that the voluntary release and withdrawal reflex can work together to reinforce heat protection. Nevertheless, fusing voluntary and reflex functions for prosthetic performance in activities of daily living awaits a more cogent strategy in sensorimotor control.

## 1 Introduction

The human sensory system is equipped with rich receptors at fingertips and vast neural circuits in the spinal cord and brain for perceiving the external environment (Johansson and Westling, 1984; Johansson and Flanagan, 2008). Nociceptors serve to protect the skin from noxious thermal stimuli by eliciting pain sensations. The nociceptive afferents inform the brain of potentially hazardous objects and initiate protective motor actions by quickly retracting the hand from the heat source, a behavior known as the withdrawal reflex (Henrich et al., 2021), resulting from the seamless sensorimotor pathways of nociceptive temperatures (Witney et al., 2004; Muniak et al., 2007; Sieck, 2019). Advanced prosthetic hands are emerging with artificial skin embedded with integrated nanosensors (Yang et al., 2019; Romeo et al., 2021; Li et al., 2022). Some flexible sensors made of materials such as hydrogel, textile, and paper could be susceptible to heat damage (Islam et al., 2020; Pinelli et al., 2020; Tai et al., 2020; Yang et al., 2021; Hardman et al., 2022; Liu et al., 2022). It is important to protect the integrity of the prosthetic hand for sustainable function since amputees may have reduced or no sensation in the residual limb, rendering them unable to perceive nociceptive temperatures or make the necessary motor adjustments for protection.

Recent research in providing temperature perception and protection in prosthetic hands has advanced significantly. Utilizing Peltier sensors, researchers have provided feedback on various temperature levels (range from 15°C to 40°C) to subjects, facilitating the perception of heat through thermal stimulation (Ueda and Ishii, 2016; Moqadam et al., 2021; Iberite et al., 2023). However, it is not clear how a nociceptive warning can be achieved. Moreover, the advent of novel sensors embedded in electronic skins has enabled prosthetic hands or robot to rapidly detect a nociceptive stimulus and to evoke protective withdrawal reflexes (Chen et al., 2022; Liu et al., 2022; Neto et al., 2022; Shi et al., 2023; Wang et al., 2023). Yet, these automatic control strategies do not involve the sensory loop with the human nociception. It remains a challenge not only to warn against harmful temperatures but also take withdraw actions via reflexive, voluntary, or hybrid control. There are two modes in which voluntary action and withdrawal reflex can work together in the human hand (Lynn et al., 2016; Henrich et al., 2021). The reinforcement control mode demonstrates the subjects’ motor intention to rush away from danger, while the contradictory mode indicates the subjects’ ability to inhibit the action of withdrawal reflex and represents that the subjects are able to tolerate this danger after careful consideration. These two control modes are both essential in practical operation in daily life. In this paper, we will focus only on the feasibility of individual control modes and a simple interactive control in which the neuromorphic withdrawal reflex reinforces voluntary release upon a nociceptive warning. Subject’s intention to inhibit the withdrawal reflex action requires careful consideration of more cognitive and behavioral factors (Ghosh et al., 2014; Lynn et al., 2016). Thus, interactive control where voluntary control may overrule the withdrawal reflex will not be investigated in this study. The objective here is to develop and verify the sensorimotor circuits for nociceptive warning and protection in prosthetic hands.

Restoring sensorimotor ability for amputees via invasive or non-invasive approaches has become an emerging technology (Bensmaia et al., 2020; Lan et al., 2023). In invasive sensory feedback technologies, intraneural electrodes including the longitudinal intrafascicular electrode (LIFE) (Dhillon et al., 2004), transverse intrafascicular multichannel electrode (TIME) (Raspopovic et al., 2014), Utah slanted electrode array (USEA) (George et al., 2020), and flat interface nerve electrode (FINE) (Tan et al., 2014) not only enable the perception of contact forces but also help amputees identify object properties, such as size, compliance, (George et al., 2020), texture (Oddo et al., 2016), and shape (D’Anna et al., 2017). Results also found that these approaches improved the amputees’ sense of embodiment, and self-confidence in manipulations of daily life (Graczyk et al., 2018; Cuberovic et al., 2019). In non-invasive technologies, alternative feedback methods based on electrotactile or mechanotactile stimulation can establish a distinct feedback mapping pathway through training and learning (Saunders and Vijayakumar, 2011; Dosen et al., 2017; Aboseria et al., 2018; Markovic et al., 2018). These alternative feedback approaches have been applied to restore the identification of objects (Akhtar et al., 2018; Dideriksen et al., 2020) and grasp control (Dosen et al., 2015; Schweisfurth et al., 2016). Compared with invasive somatotopic sensory feedback approaches, these alternative approaches showed less robustness and high cognitive process in the manipulation tasks (Valle et al., 2020). Transcutaneous electrical nerve stimulation (TENS) on the skin surface can induce somatotopic sensations that do not require complicated implant surgery or additional training. Using TENS based on evoked tactile sensations (ETS), amputees were able to stably perceive information such as forces and apertures (Chai et al., 2015; Zhang et al., 2022b). The finger-specific sensations have become a promising approach for amputees to provide a sensory foundation for noxious temperatures (Hao et al., 2020). In this study, we applied TENS to restore the perception of forces and nociception.

There were four specific aims in this study. First, a rapid algorithm was validated to predict the steady-state temperature from the slow response of a thermosensitive sensor to establish further thermal warning action timely. Second, a hybrid sensory coding strategy was designed to allow the delivery of prosthetic contact forces and nociceptive heat warnings via TENS to evoke dangerous awareness and voluntary protection. Third, a biomimetic withdrawal reflex control strategy was implemented on a tendon-driven prosthetic hand with antagonistic neuromorphic muscle models to reinforce voluntary release. Finally, the closed-loop system was tested and verified in able-bodied and amputee subjects to demonstrate the feasibility of providing nociceptive warning and protection in both a voluntary and a reinforcing manner.

## 2 Methods and materials

### 2.1 Subjects

**Figure 1:**
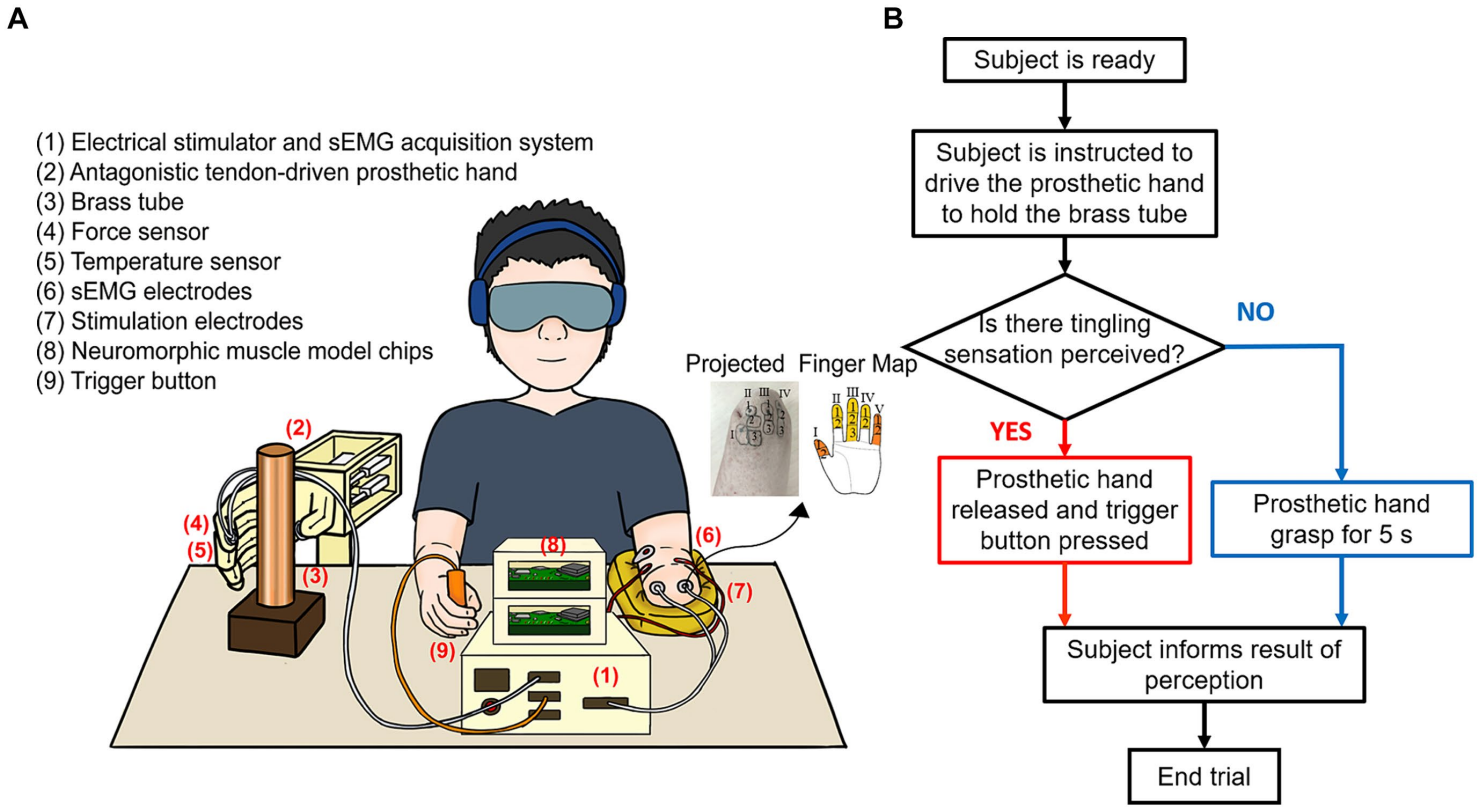
Experimental protocol and workflow diagram. **(A)** The antagonistic tendon-driven prosthetic hand was anchored to the table. Subjects activated their sEMG signals to grasp a tube containing water of different temperatures after receiving the instructions from the experimenter. **(B)** Test flowchart showing subject responded differently based on perception.

A total of six subjects were recruited, including four able-bodied individuals (H1–H4, 2 males and 2 females, 26 ± 2.0 years old) and two male forearm amputees with intact projected finger maps as shown in Figure 1A (A1–A2, 62 ± 2.0 years old). The study was approved by the Ethical Committee of Human and Animal Studies at Shanghai Jiao Tong University (E2020021I), and all subjects were informed of the experimental procedures and signed an informed consent form before the experiments. Two able-bodied subjects (H1–H2) and two amputees participated in Test 1 and all six subjects participated in Test 2 due to variation.

### 2.2 Experimental setup

The experimental setup was illustrated in Figure 1A: (1) an electrical stimulator and a sEMG acquisition system, (2) an antagonistic tendon-driven prosthetic hand, (3) a brass tube, (4) force sensors (Flex Force A201, Tekscan, United States), (5) a thermosensitive temperature sensor (LMT70YFQT, Texas Instruments, United States), (6) customized sEMG electrodes, (7) customized stimulation electrodes, (8) neuromorphic muscle model chips (Spartan-6, Xilinx, United States), and (9) a trigger button.

A tendon-driven prosthetic hand (2) controlled by a pair of antagonistic muscle models was used in this study (Xie et al., 2022), which was modified from a 3D-printed open-source hand (InMoov, 2012, France). This prosthetic hand was fixed to a table, and a highly thermally conductive brass tube (50 mm in diameter, 1 mm in thickness, 250 mm in height) was in front of it (3). A temperature sensor (5) was placed on the index finger, and two force sensors (4) were placed on the index and thumb fingers. Two steel cables with a radius of 0.5 mm were passed through and fixed to the medial and lateral sides of a finger to mimic the finger extensor and flexor. Each cable was connected to a linear motor (LAF 30, Inspire Robotics Inc., Beijing, China), and two linear motors drive a finger through the position-force proportional and integral control in extension and flexion motions within a single degree of freedom. Two motors decoded the target muscle forces corresponding to the antagonistic muscle models. The maximum grip force in a single finger was 15 N. The antagonistic muscles were implemented by two Hill muscle models, respectively, (Shadmehr and Wise, 2005; Mileusnic et al., 2006). The muscle force was generated by the real-time computational model via a neuromorphic chip (8) (Niu et al., 2014, 2017). Muscle contraction force was mainly calculated by alpha motor commands based on a force-alpha motor command relationship (Cheng et al., 2000; Mileusnic et al., 2006; Song et al., 2008). Subjects used their sEMG signals to grasp the tube with a pinch grip as shown in Figure 1B. Electrical stimulator and sEMG acquisition system (1) acquired sEMG signals and implemented a multi-channel, programmable TENS sensory stimulation function. Since sEMG signals and electrical stimulation signals may interfere with each other, we used hardware blanking and software digital filtering to eliminate the artifacts, and two channels of sEMG signals (6) were sampled in 2 kHz (Yu et al., 2022). This system also acquired signals from two channels of force sensors, one channel of temperature sensor, and one channel of trigger button, and converted the corresponding force and temperature signals into multi-channel stimulation pulses, which were transmitted to the projected finger map area of the amputee through customized Ag/AgCl stimulation electrodes (7) to evoke tactile sensations (Zhang et al., 2022b). Stimulation currents were biphasic, charge-balanced, cathode-first pulse trains with a 10 μs interval between pulses. Force and temperature signals were collected with a sampling frequency of 100 Hz and a trigger button (9) was used to mark the perceptual events. In this study, normalized sEMG was used to represent the alpha motor command, with the sEMG normalized to 0 and 1 when the subject was resting and exerting maximum voluntary contraction force, respectively. More detailed information was published in previous research (Xie et al., 2022; Zhang et al., 2022b, Zhang Z. et al., 2022c).

### 2.3 Steady-state temperature prediction algorithm

**Figure 2:**
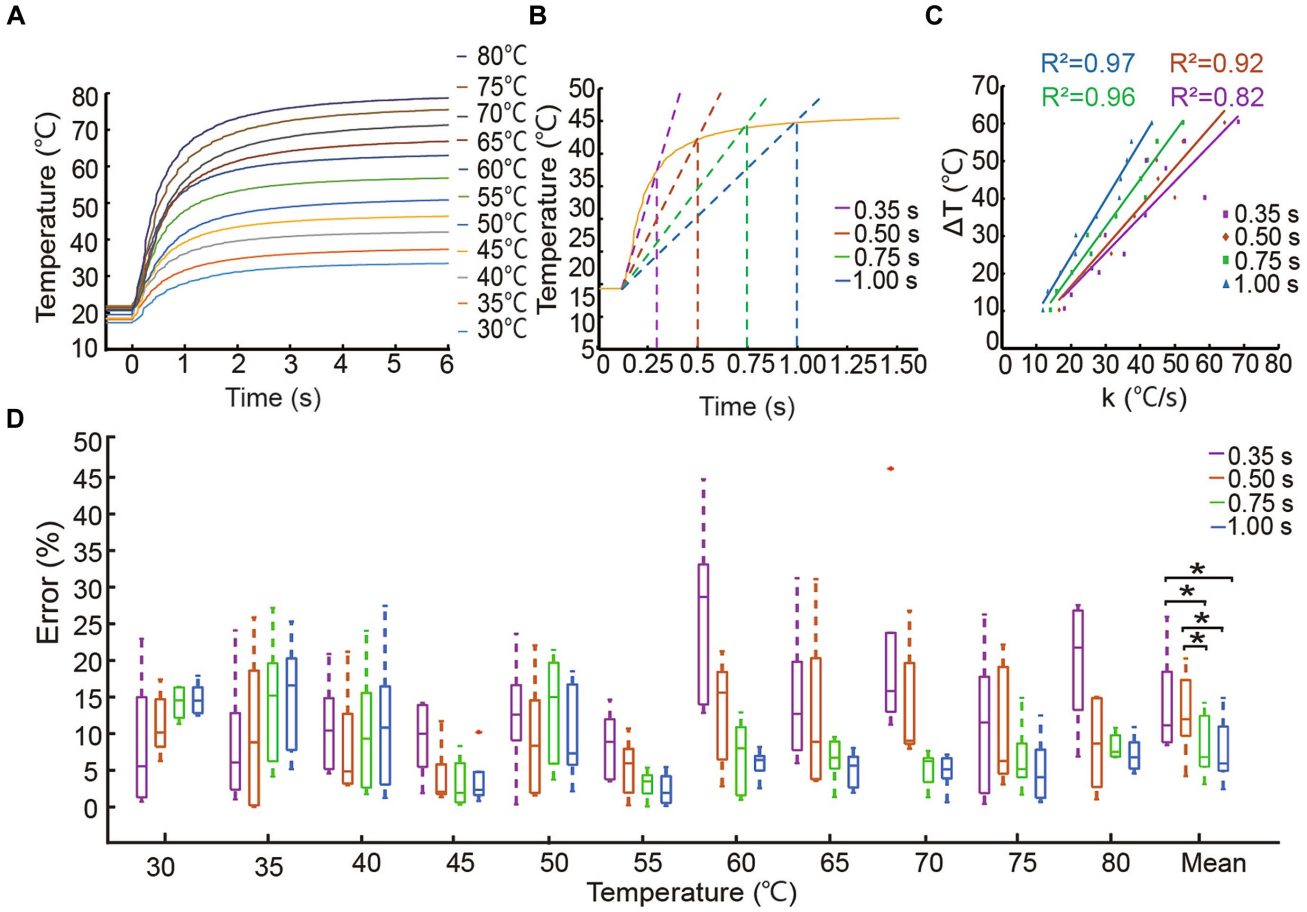
Accuracy of steady-state temperature prediction algorithm. **(A)** Response curves of the temperature sensor measuring thermostatic plate at different temperatures. **(B)** Prediction algorithm diagram at 45.0°C based on four identification times. **(C)** The *k* − Δ*T* relationship curve. **(D)** The errors of the prediction algorithm correspond to four identification times with *p < 0.05.

Temperature sensors exhibit various slopes of voltage output at different temperatures. Typically, the thermosensitive temperature sensor we utilized in this study requires a response time of 6 s to detect a steady-state temperature as shown in Figure 2A. Such a long response time does not allow for the timely detection of nociceptive temperatures that could pose a danger. To shorten the response time of the temperature sensor and provide timely heat warnings, we developed a steady-state temperature prediction algorithm based on the voltage slope of the sensor. The temperature sensor was calibrated by placing it on a thermostatic heating plate (DB-XAB, Lichen Bangxi Instrument Inc., China) for 10 s to establish the relationship between output voltage and constant temperature, ranging from 30.0°C to 80.0°C with a step of 5.0°C. Ten repeat measurements were taken for each temperature.

The temperature calibration and tests were carried out at room temperature, and the initial temperature was set at 20.0°C. We recorded the temperature at initial contact ($T_{\text{initial}\;\text{state}}$) and steady-state after contact for four fixed sliding times ($T_{\text{steady}\;\text{state}}$). Then, a Savitzky–Golay filter was used to calculate the slope ($k_{\text{initial}\;\text{state}}$) of sensor voltage at $T_{\text{initial}\;\text{state}}$ for a fixed sliding time (Kordestani and Zhang, 2020), which was defined as the identification time. We chose four identification times including 0.35 s, 0.50 s, 0.75 s, and 1.00 s. Based on the relationship of the temperature difference (ΔT) between the initial-state and steady-state, and the initial slope ($k_{\text{initial}\;\text{state}}$) using Eq. 1, a linear relationship could be used to predict the steady-state temperature as shown in Eq. 2, where a and b coefficients were obtained by linear fitting of initial slope and temperature difference under four sliding time lengths. Prediction error was defined as the percentage of absolute error between the actual temperature set on the thermostatic plate and the temperature predicted by the algorithm. In this study, the nociceptive temperature threshold ideally considered both amputee safety and prosthetic material limitations and could be set based on the lower of the two limits. Building upon the understanding that the TRPV2 ion channel in human nociceptors triggers pain sensations at 52.0°C (Wang et al., 2023), this study set a hypothetical noxious temperature warning threshold at 60.0°C as a proof of concept.


(1) $$\Delta T=ak_{\text{initial}\;\text{state}}+b$$

(2) $$T_{\text{steady}\;\text{state}}=\Delta T+T_{\text{initial}\;\text{state}}$$

### 2.4 Hybrid sensory strategy

Previous research has verified that ETS elicited by TENS restored digit-specific sensations for amputees via the direct neural pathway (Hao et al., 2020). The stability of the ETS location and area, as well as the sensory thresholds for each sensation were also verified (Chai et al., 2015). Tingling sensation was evoked by high frequency or amplitude and was described as a tolerable tingling sensation but no pain and no muscle contraction (Amer-Cuenca et al., 2011; Johnson et al., 2015). The characterization of tingling could be used to encode the nociceptive warning to indicate that prosthetic hand was touching something hot. Buzz sensation was described as a strong and continuous high frequency vibration and has been shown a wide pulse width modulation range and high sensitivity (Chai et al., 2015; George et al., 2019; Zhang et al., 2022a), it was chosen to encode force information.

We identified our participants’ perceived locations of their fingers on their residual limb using a 2 mm diameter metal pen with a rounded tip and placed stimulating electrodes over these areas. Stimulation frequency was set at 50 Hz. Amplitude and pulse width ranges were customized for each subject based on the rapid assessment strategy developed in our previous study (Yang et al., 2020; Zhang et al., 2022b). In this protocol, the amplitude was increased from an initial value of 1 mA in 0.5 mA step until the buzz or tingling sensations were first clearly perceived. Under the determined amplitude, the pulse width was initially set at 20 μs and gradually increased in 20 μs step. The minimum pulse width was defined as the sensory threshold when the buzz or tingling modality was first clearly perceived ($W_{\text{buzz}:\min}$,$W_{\text{tingling}:\min}$), and the maximum pulse width was defined as the value at which the sensory modality began to change ($W_{\text{buzz}:\max}$). Each electrical stimulus used to assess the sensory threshold had a duration of 3 s, with a rest period of 5 s before the next stimulus. The stimulation parameters of all subjects for these two sensory modalities were summarized in Supplementary Table S1. The maximum ($F_{\text{max}}$) and minimum grip force ($F_{\text{min}}$) generated by subjects when grasping the tube were tested and mapped to pulse width using Eq. 3. To reduce discomfort for the subjects, a constant pulse width was encoded using the sensory threshold at which the tingling sensation first appeared, as shown in Eq. 4. When the temperature sensor did not detect a nociceptive temperature, the stimulation of the index and thumb stimulation sites was a buzz modality. As soon as a harmful temperature was detected, stimulation parameters were immediately switched to the tingling modality in the index finger stimulation site, while stimulation of the thumb finger was paused.


(3) $$W=\left\{ \begin{array}{c} 0\;\text{if}\;F<F_{\text{min}}\\ \frac{W_{\text{buzz}:\max}-W_{\text{buzz}:\min}}{F_{\text{max}}-F_{\text{min}}}*\left(F-F_{\text{min}}\right)+W_{\text{buzz}:\min}\;\text{if}\;F_{\text{min}}\leq F<F_{\text{max}}\\ W_{\text{buzz}:\max}\;\text{if}\;F\geq F_{\text{max}} \end{array}\right.$$

(4) $$W=W_{\text{tingling}:\min}\;\text{if}\;F\geq 0.1\;N\;\text{and}\;T\geq 60.0^{{}^{\circ}}C$$

### 2.5 Withdrawal reflex control strategy

Neuromorphic muscle control technology mimics the biomechanical properties of the human hand through calculations involving motor neurons and muscle models. Experimental evidence has demonstrated that this method has significantly superior control results compared to traditional proportional control methods (Niu et al., 2021; Luo et al., 2021a,b). To reconstruct the control loop of nociceptive temperature, we designed a neuromorphic withdrawal reflex circuit.

In scenarios where the detected temperature was below 60.0°C, the bionic withdrawal reflex was not triggered, and no reflex compensation was superimposed on the alpha motor commands. Once the prosthetic hand grasped an object (F≥0.1N) and the temperature sensor predicted a steady state temperature exceeding 60.0°C, the withdrawal reflex circuit was activated. The voltage output of the temperature sensor was converted into the presynaptic current ($I_{ncp}$) to excite the interneuron. We utilized the Izhikevich neuron model to simulate the interneuron, calculating a binary spike train, membrane potential ($V_{\text{itn}}$), membrane recovery variable ($U_{\text{itn}}$) induced by a nociceptive temperature (Izhikevich, 2003), as shown in Eqs. 5, 6.


(5) $$\left\{ \begin{array}{c} {V_{\text{itn}}}^{\prime }=0.04{V_{\text{itn}}}^{2}+5V_{\text{itn}}+140-U_{\text{itn}}+I_{ncp}\\ {U_{\text{itn}}}^{\prime }=a\left(bV_{\text{itn}}-U_{\text{itn}}\right)\; \end{array}\right.$$

(6) $$\text{if}\;V_{\text{itn}}\geq 30\text{mV},\text{then}\;\left\{ \begin{array}{c} V_{\text{itn}}\leftarrow c\\ U_{\text{itn}}\leftarrow U_{\text{itn}}+d \end{array}\right.$$

**Figure 3:**
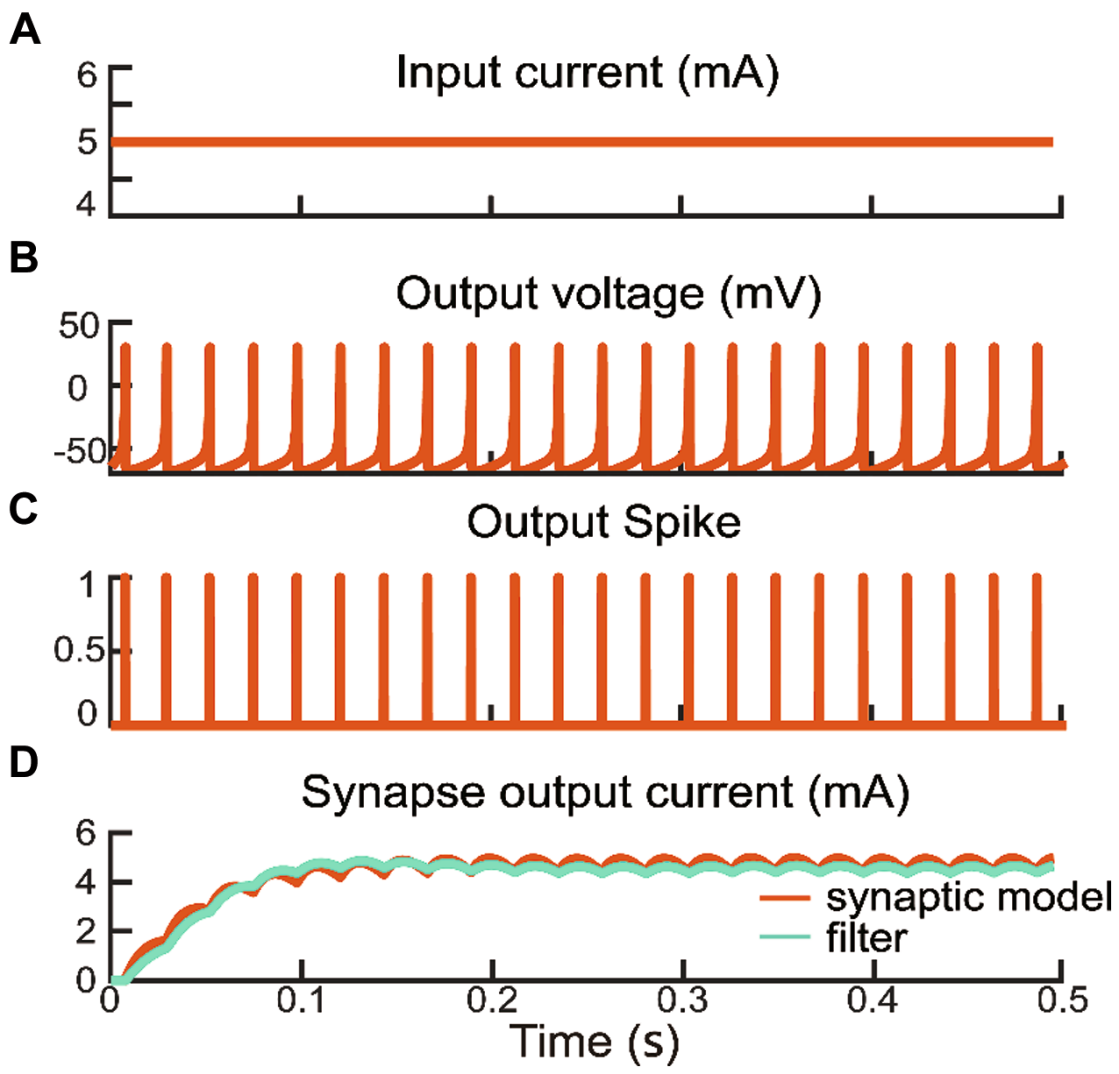
A constant current input from interneuron layer generated a series of spike to synapse layer including model and filter outputs. The parameters of interneuron model were as following: *a* = 0.1 ms^−1^, *b* = 0.2 ms^−1^, *c* = −65 mV, *d* = 2 mV/ms.

Upon reaching the firing threshold, the excitatory interneuron generated the postsynaptic current ($I_{\text{itn}}$). We adjusted the rise and fall time parameters of the synaptic model ($\tau_{r}=0.08\;s,\tau_{f}=0.19\;s,V_{m}=5.2\;\text{mV}$) (Glowatzki and Fuchs, 2002; Niu et al., 2017), as delineated by Eq. 7. The excitatory postsynaptic current also activated an inhibitory interneuron, which generated an inhibitory postsynaptic current (Li et al., 2015). The excitatory postsynaptic current served as reflex compensation to the extensor alpha motor command, while the inhibitory postsynaptic current was directed to the flexor alpha motor command. Consequently, the biomimetic withdrawal reflex response was manifested by a reduction in flexor activity preventing further grasping, and an increase in extensor activity facilitating a rapid release of the grip. These synaptic computations were realized through filtering by a host computer. To validate the consistency between the postsynaptic current generated by the filter and the synaptic model, a constant current was input into the interneuron model (Figure 3A). After the interneuron model consistently produces the membrane potential and a binary spike train as shown in Figures 3B,C, the synaptic model begins to compute postsynaptic current as shown in Figure 3D. If the interneuron is not activated or is not activated enough to produce a binary spike train, the synaptic current will be 0.


(7) $$I_{\text{itn}}\left(t\right)=\left\{ \begin{array}{c} V_{m}\left(e^{-\frac{t}{\tau_{f}V_{m}}}-e^{-\frac{t}{\tau_{r}V_{m}}}\right),\text{if}\;t\geq 0\\ 0,\text{otherwise} \end{array}\right.$$

### 2.6 Experimental protocols

We first validated the feasibility of the neuromorphic withdrawal reflex control strategy without subject participation. Based on the validation of reflexive heat protection, Test 1 and Test 2 with the subjects’ participation were designed to investigate how similar neuromorphic mechanisms implemented in prosthetic hands could allow amputees to release the hot object upon a nociceptive warning in voluntary and reinforcement mode. Subjects were not informed of the experimental conditions during the two tests.

The validation of the neuromorphic withdrawal reflex in the prosthetic hand under no-load condition was executed by a host computer that directly issued constant alpha motor commands (*α* = 0.7 for the flexor, *α* = 0.2 for the extensor). Building upon the verification of temperature prediction accuracy, we tested the prosthetic hand grasping a tube at two temperatures: one above the warning threshold at 65.0°C and another below the threshold at 30.0°C, each repeated 40 times. If the predicted temperature exceeded the warning threshold, the biomimetic withdrawal reflex circuit was triggered. Interneurons and synapses calculated the excitatory postsynaptic current, which was then superimposed onto the extensor alpha motor neuron as reflex compensation. Conversely, a negative reflex compensation value decreased the flexor alpha motor command.

Test 1 aimed to validate the feasibility of providing nociceptive warning and voluntary protection through a psychophysical experiment (Osborn et al., 2018; Valle et al., 2018). Water at nine different temperatures was placed inside the tube (30.0°C, 40.0°C, 50.0°C, 55.0°C, 57.5°C, 62.5°C, 65.0°C, 70.0°C, and 80.0°C). Each temperature was tested in ten trials and randomly assigned during the test. Subjects used sEMG signals to grasp the tube under instruction. Subjects’ auditory and visual senses were occluded with earmuffs and eye masks. The experimenter signaled the start and end of each trial by tapping the subject’s shoulder. A buzz sensation indicated the water was below the warning threshold, prompting subjects to maintain their grasp for 5 s until a subsequent shoulder tap. A tingling sensation indicated water above 60.0°C, signifying potentially harmful heat and necessitating an immediate release of the grasp by the prosthetic hand, while the subjects were asked to press a button with their contralateral hand to record perception time as shown in Figure 1B. Data were provided by only two able-bodied individuals (H1, H2) and two amputees (A1, A2) due to data collection constraints.

The purpose of Test 2 was to test the integration of the withdrawal reflex strategy with the hybrid sensory strategy and to validate the feasibility of providing nociceptive warning and protection in a reinforcement way. The setup and requirements for Test 2 were identical to those of Test 1. Similarly, the test involved two temperatures set at 65.0°C and 30.0°C, with each temperature undergoing ten repeated trials. Once the predicted temperature exceeded the warning threshold, the biomimetic withdrawal reflex circuit was activated, switching the stimulation modality to the nociceptive temperature warning. To confirm the reinforcement of voluntary release and withdrawal reflex, subjects were instructed to voluntarily open the prosthetic hand and press a button, aligning their actions with the reflex response upon perceiving the tingling sensation. If a tube heated at 30.0°C was grasped, subjects were asked to maintain the grasp for 5 s upon perceiving the buzz sensation. All participants took part in Test 2.

### 2.7 Data processing and statistical analysis

All control programs were written in C# language and ran on the Visual Studio 2019 platform. Data processing was completed using Visual Studio Code 2020 in Python. In Test 1, the probability of perceiving the tingling sensation after ten trials was statistically computed for each temperature setting. A sigmoid function was used to fit the quantitative relationship between the tested temperatures and the probabilities of nociceptive temperature perception, revealing subjects’ ability to perceive nociceptive temperature warnings via a hybrid sensory strategy. The 50% probability point of the fitted curve, known as the point of subjective equality (PSE) (Valle et al., 2018), represented the subjective nociceptive temperature warning threshold perceived through the sensory strategy. We defined the following performance indices to measure the subjects’ perception of nociceptive temperature warnings and the speed of their response. The time required for sliding window of the temperature prediction algorithm to output a response was defined as the identification time (IT). Action time (AT) was defined as the time interval from grasping the tube to the change in alpha motor commands. Perception time (PT) was determined as the time interval from when the steady-state temperature was predicted to when the trigger button was pressed. Execution time (ET) was defined as the time interval from when the heat warning temperature was predicted to when the index fingertip force disappeared. Reaction time (RT) was the total time from grasp to release and was equal to the sum of the IT and ET. With reference to Figure 2D, the boxes indicated the interquartile range (IQR), the line within the boxes depicted the median, and the whiskers represented maximum and minimum values of the performance index for subject participation in each test. No dataset passed the one-sample Kolmogorov–Smirnov test, which indicated that all datasets were not normally distributed. The Wilcoxon signed rank test with Bonferroni correction for nonparametric tests was performed on pairwise comparison. The results of all tests were also analyzed in between-subjects comparison, and there were not significantly different. Horizontal continuous lines denoted statistically significant differences in the dispersions. All data were combined for statistical analysis using IBM SPSS Statistics 26.0.

## 3 Results

### 3.1 Accuracy of temperature prediction algorithm

Time-temperature response curves are shown in Figure 2A. Figure 2B used the example of 45.0°C to illustrate how steady-state temperature could be predicted with four different identification times based on slopes. Figure 2C showed that all the initial slopes were strongly correlated with the difference between the steady-state and initial-state temperatures under four identification times. Figure 2D depicted prediction accuracy with four identification times in each temperature block for ten trials. Prediction errors were low across the range of tested temperatures with average errors of 13.68 ± 10.19%, 11.00 ± 8.70%, 8.30 ± 6.00%, and 7.70 ± 6.20%. The average errors of the lower identification times (0.35 s and 0.50 s) were significantly different than those associated with the higher identification times (0.75 s and 1.00 s), and there was no significant difference between 0.75 s and 1.00 s. Considering prediction speed and accuracy, we selected 0.75 s as the identification time for subsequent tests. These results illustrated the robustness and accuracy of this algorithm, which was much faster than the sensor’s response time and contributed to the fast and safe detection of nociceptive temperatures.

### 3.2 Performance of withdrawal reflex strategy

**Figure 4:**
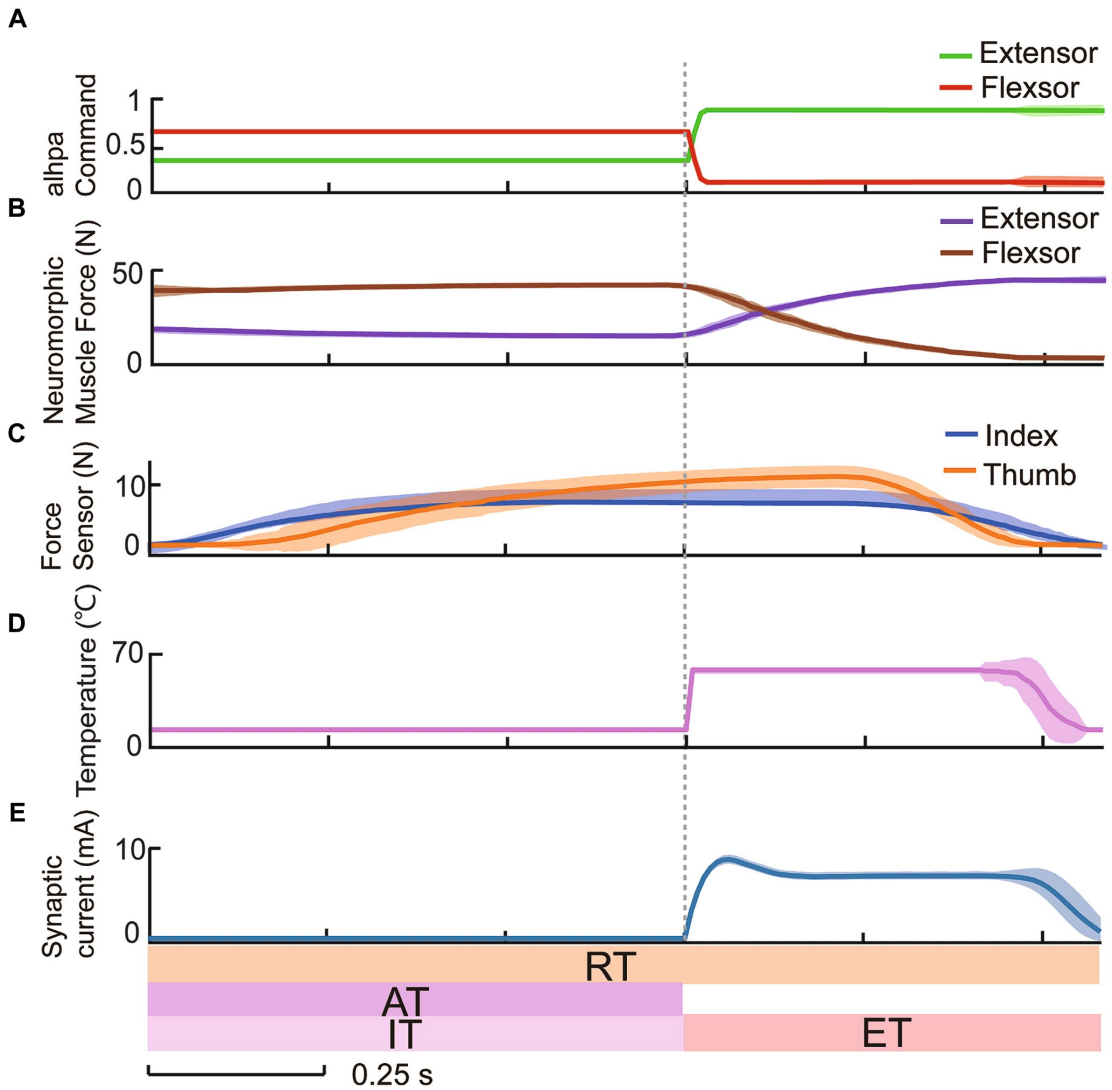
Constant alpha commands controlled by host computer demonstrated the reflexive protection to 65.0°C temperature.

The response of the withdrawal reflex circuit was evaluated. With constant activation of alpha command in Figure 4A, the two servo motors followed the antagonistic muscle forces computed by the neuromorphic muscle models (Figure 4B) and grasped the tube as shown in the fingertip force trajectories (Figure 4C). After the identification time, the temperature exceeded the warning threshold in Figure 4D, the interneuron and synapse models produced synaptic currents (Figure 4E). The synaptic currents inhibited the flexor muscle and excited the extensor muscle immediately. This action caused the hand to open, thus releasing the grasped tube during the execution time. If the temperature was below the warning threshold, the withdrawal reflex control loop remained inactive, with alpha command, neuromorphic muscle forces, and contact forces all remaining unchanged and no synaptic output. These results demonstrated the bionic withdrawal reflex’s ability to tune alpha command while detecting noxious temperatures under system no-load condition, verifying the feasibility of reconstructing a bionic withdrawal control loop using neuromorphic muscle control technology in the prosthetic hand.

### 3.3 Performance of hybrid sensory strategy

**Figure 5:**
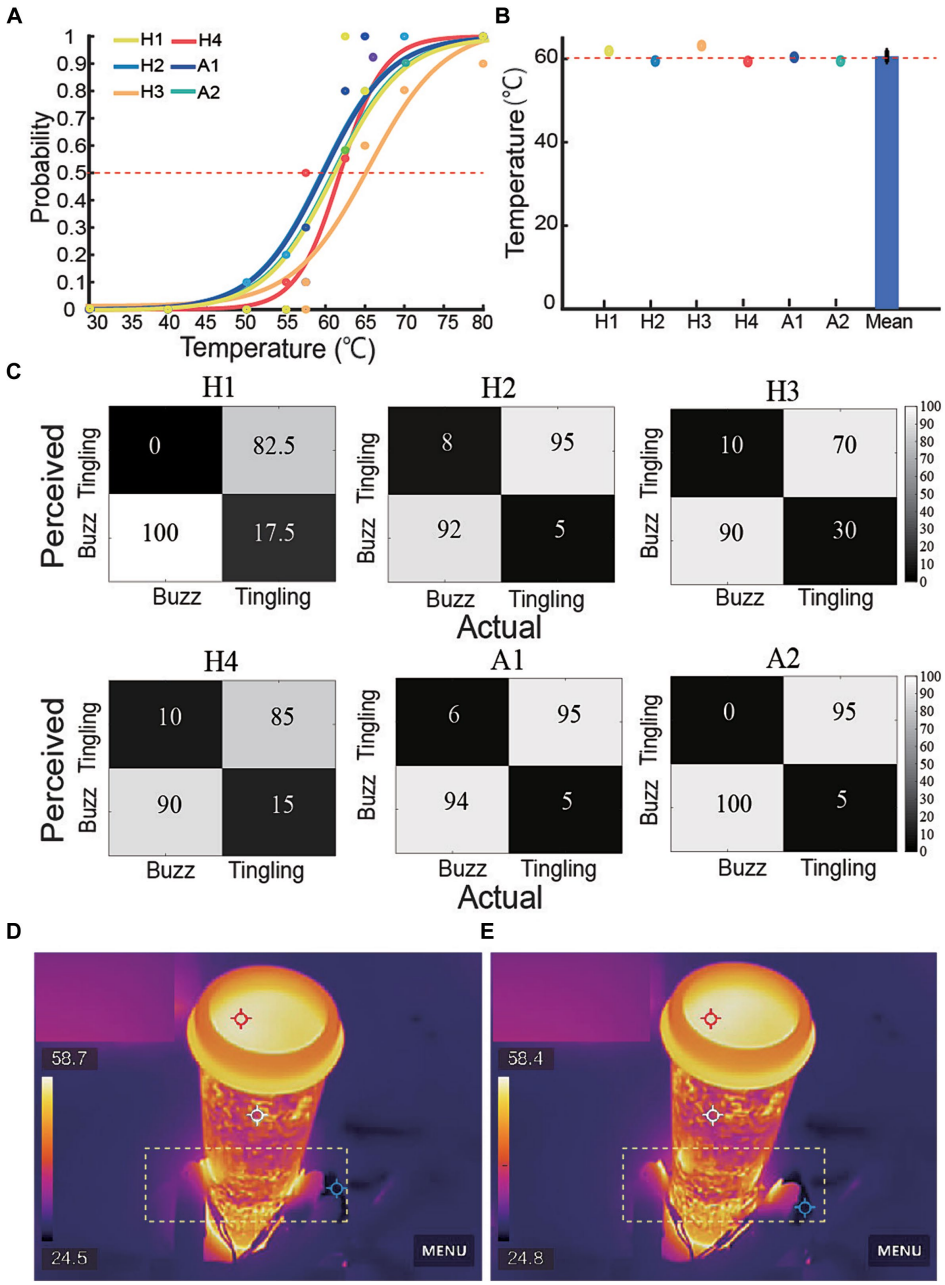
Perception response to nociceptive stimulation of four able-bodied and two amputee subjects. **(A)** Nociceptive perception probability curves in all subjects, red line represented the 50% probability. **(B)** Point of subjective equality (PSE) for each subject’s perception of nociceptive temperatures compared to the internally set heat warning threshold in red line (*p* > 0.05). **(C)** Confusion matrices represented all subjects’ identification accuracy of buzz and tingling sensations. **(D,E)** Infrared imaging of grasping and releasing of tube with hot water. The yellow dotted box highlighted the grasp and release actions.

Figure 5A illustrated the fitted psychophysical curves based on response probability for a range of temperature blocks from 30.0°C to 80.0°C in four able-bodied and two amputee subjects. The horizontal axis was the tested temperature and the vertical axis was the probability of perceiving a tingling sensation at each tested temperature. The 50% probability point of the fitted psychophysical curves were the PSE for each subject, which could demonstrate the nociceptive perception ability. Figure 5B showed that the PSE for each subject’s perception of harmful temperatures was not significantly different from each other. The average PSE in the nociceptive temperature threshold was 61.3°C, which was close to the warning threshold internally set at 60.0°C represented by a red horizontal line. Figure 5C presented confusion matrices for the recognition of buzz and tingling sensations with the six subjects achieving average success rates of 86.90 and 94.30%. Figures 5D,E presented the infrared imaging of a subject controlling a prosthetic hand to hold the tube with hot water and release the tube, which was highlighted in the yellow dotted box.

These results suggested that despite individualized hybrid sensory coding strategies, amputees were able to distinctly perceive the warning of nociceptive temperatures and force. These results also demonstrated that a hybrid sensory strategy could provide feed-forward sensory commands for subsequent adjustment of protection motor commands.

### 3.4 Nociceptive warning and voluntary protection

**Figure 6:**
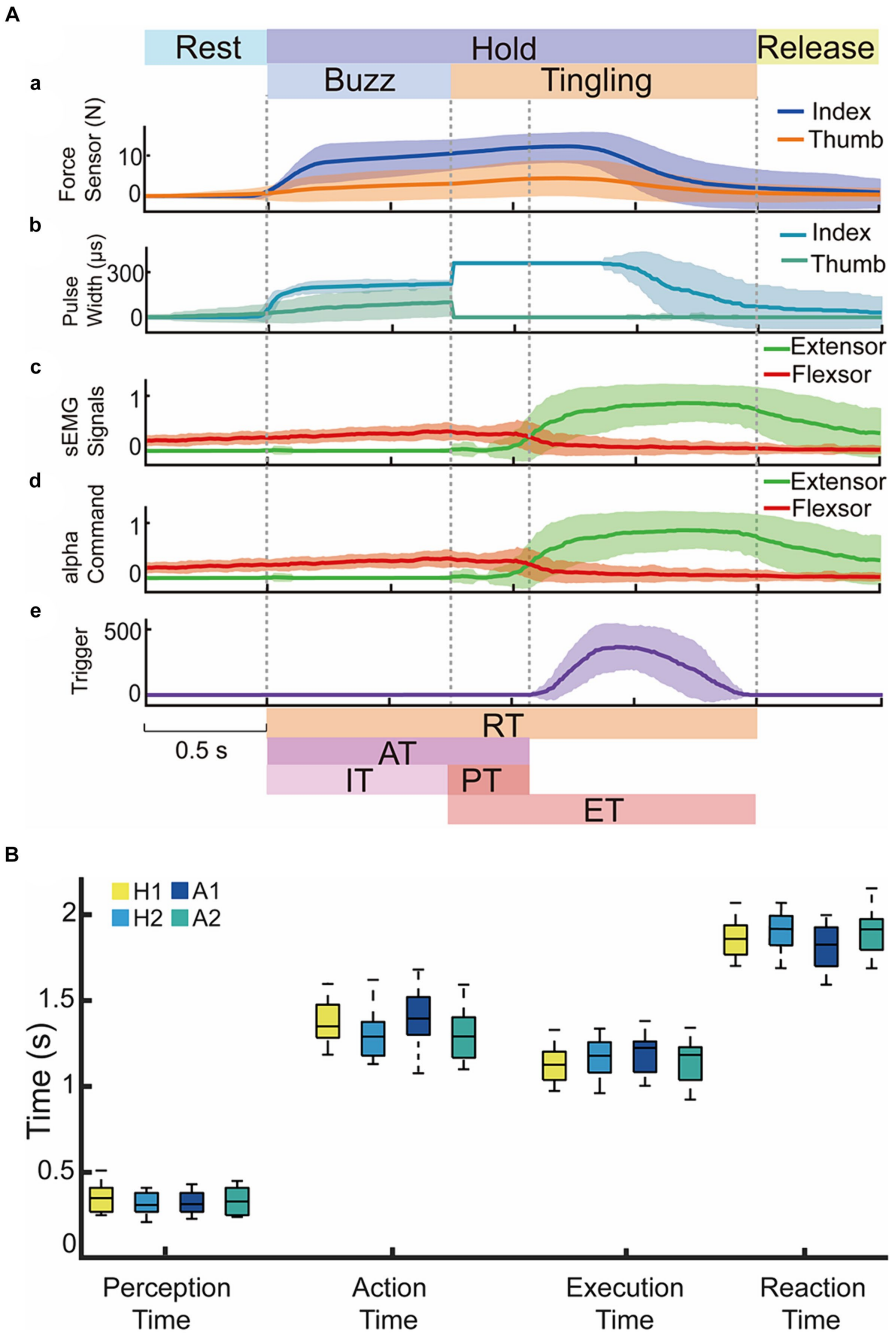
The time responses in Test 1 for voluntary opening actions to four nociceptive temperatures at 62.5°C, 65.0°C, 70.0°C, 80.0°C. **(A)** Representative data from subject H2 for contact force, stimulus pulse width, sEMG signals and alpha motor commands. Dark lines represented the average values and light shadow area represented mean ± standard deviation. **(B)** Performance indices of voluntary responses for two able-bodied and two amputees in Test 1.

Figure 6A depicted the representative complete process of one subject (H2) volitionally controlling the sEMG to open the prosthetic hand upon perceiving a tingling sensation at 62.5°C, 65.0°C, 70.0°C, 80.0°C. The subject grasped the tube using the flexor sEMG as shown in Figure 6A(a). After an identification time of 0.75 s, the detected temperature was above the warning threshold, the system switched from the buzz stimulus modality to the tingling stimulus modality (Figures 6A(b)). The subject increased the extensor sEMG significantly (Figures 6A(c,d)) after the perceived tingling sensation (Figures 6A(e)), leading to a gradual reduction in prosthetic hand force and ultimately opening the hand. Figure 6B showed four performance indices for H1, H2, and A1, A2. The time indices of AT, PT, ET, and RT showed no significant difference between each subject (*p* > 0.05). Test 1 confirmed the feasibility of reconstructing the sensory circuit for noxious temperatures in a prosthetic hand using TENS based on ETS technology, amputees could voluntarily control the opening of the prosthetic hand after perceiving a tingling warning sensation.

### 3.5 Nociceptive warning and reinforcement protection

**Figure 7:**
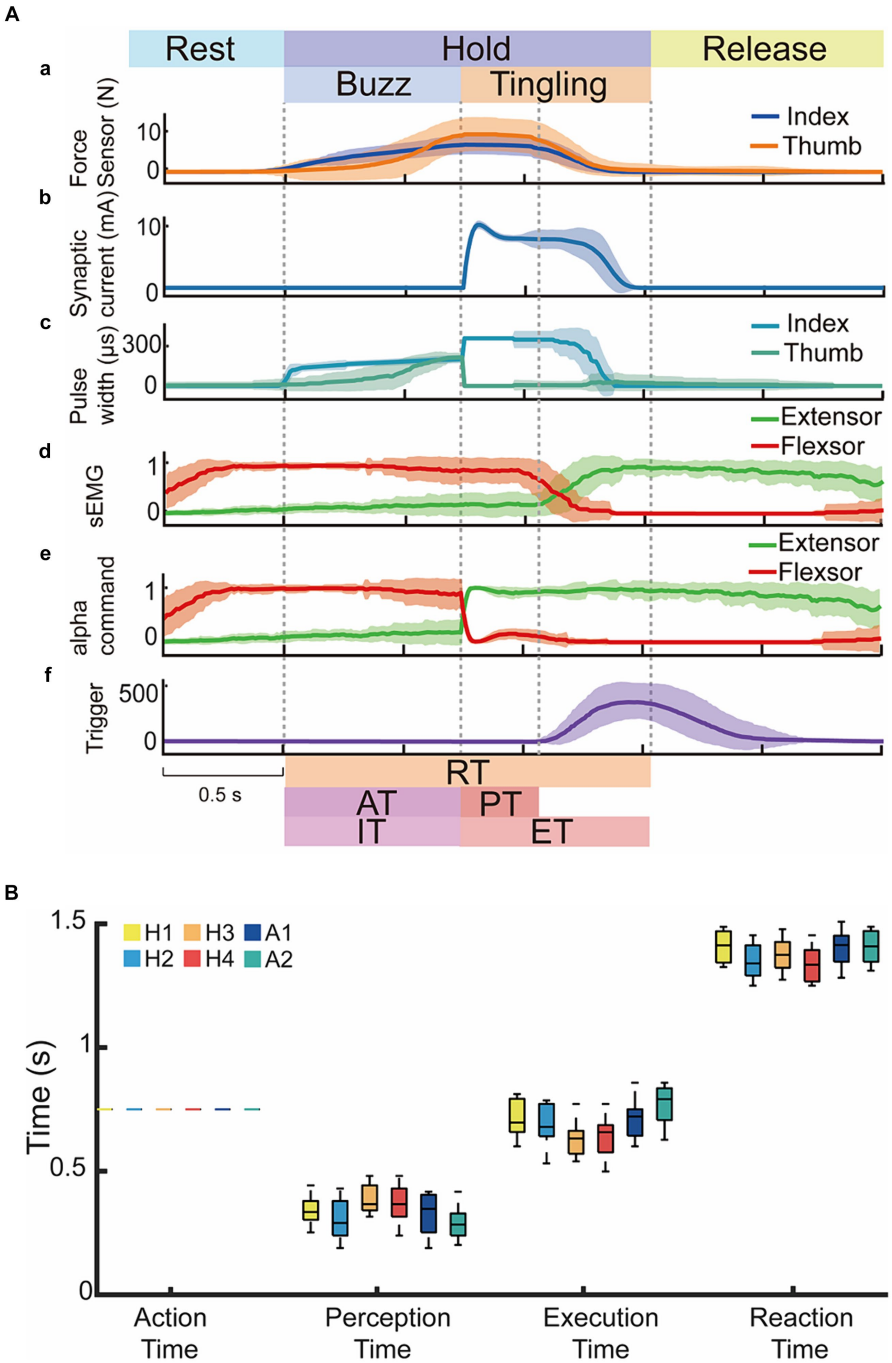
A total of six subjects (four able-bodied subjects and amputees) performed Test 2 in the cooperated reflex and voluntary control at 65.0°C nociceptive temperature. **(A)** Representative data from subject H2 to nociceptive heat stimulation with voluntary action and withdrawal reflex. Dark lines represented the average values and light shadow areas represented mean ± standard deviation. **(B)** Performance indices of six subjects in Test 2.

Test 2 demonstrated the results of the joint operation between the withdrawal reflex control strategy and the hybrid sensory strategy. This representative process from subject H2 was described in Figure 7A, where the changes in sEMG trailed but strengthened these in alpha motor commands following the nociceptive warning as shown in Figure 7A(c–e). This was demonstrated as soon as the synaptic current was firing (Figure 7A(b)), and the extensor motor command immediately reached the maximum value from excitatory action and the flexor motor command disappeared due to the inhibitory effect. The sEMG signals remained with no significant changes until the subject perceived a tingling sensation (Figure 7A(f)). Release of hand was indicated by the disappearance of fingertip forces (Figure 7A(a)). This showed that the nociceptive withdrawal reflex could accelerate the protection process of the prosthetic hand in addition to the subject’s voluntary action for the first time. There was no significant difference between four able-bodied and two amputee subjects in the performance of AT, PT, ET, and RT as shown in Figure 7B.

Test 2 proved the effectiveness of reinforcement of voluntary release and withdrawal reflex, thus verifying the feasibility of reconstructing a sensorimotor closed-loop for noxious temperature in a prosthetic hand and restoring the amputee’s ability to warn and shield against harmful temperature.

### 3.6 Time index analysis

**Figure 8:**
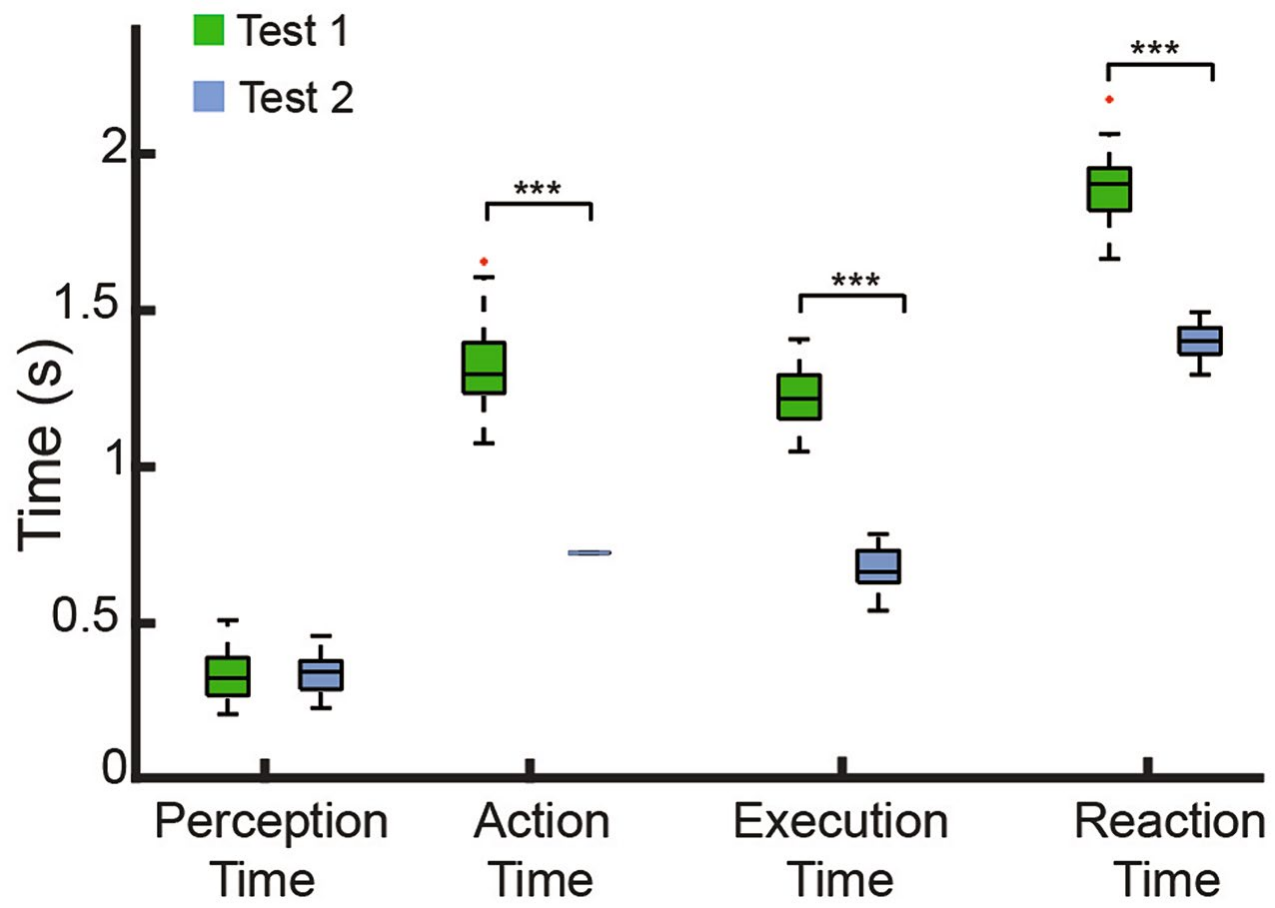
Comparison of the time indices observed in the two tests. There was a significant difference in the AT, ET and RT between Test 1 and Test 2, suggested the effectiveness of the biomimetic withdrawal reflex in expediting the motor response, ***p < 0.001. No significant difference in PT was found between Test 1 and Test 2, both of which involved participant engagement.

With the involvement of the bionic withdrawal reflex, the action time of Test 2 was equal to the identification time (0.75 s) as shown in Figure 8, and was significantly faster than the action time of Test 1 with a median point of 1.32 s (IQR: 0.16), which demonstrated that the bionic withdrawal reflex was able to move away from the nociceptive temperatures in the first instance. Perception time belonged to the subjects’ sensory ability, so there was no significant difference between Test 1 (median point: 0.33 s, IQR: 0.12) and Test 2 (median point: 0.35 s, IQR: 0.09). The execution time that the prosthetic hand opened at the first time with maximal force in Test 2 (median point: 0.62 s, IQR: 0.07) was significantly faster than in Test 1 (median point: 1.19 s, IQR: 0.14), which required subjects to voluntarily increase the extensor muscle force to open. Similarly, Test 2 (median point: 1.39 s, IQR: 0.05) was still significantly faster than Test 1 (median point: 1.93 s, IQR: 0.13) in terms of reaction time. These results could be explained by the following neurophysiological properties of the human hand. The volitional control commands induced by sEMG go through the brain to make decisions belonging to the voluntary control, while the withdrawal reflex was equivalent to the short-latency spinal reflex, which prioritized the occurrence of the voluntary control to protect the human hand in the fastest way after sensing nociception (Andersen, 2007; Zangrandi et al., 2021). Results further illustrated that our study reconstructed a natural stereotypical sequence of hand activity in prostheses, effectively restoring the amputee’s capacity for both warning and protection against nociceptive temperatures.

## 4 Discussion

Perceiving nociceptive sensations and avoiding potential dangers for amputees is valuable, but it is not clear whether a nociceptive temperature warning and protection could be achieved in current prosthetic hand solutions (Bensmaia and Miller, 2014; Manz et al., 2022). In response to this need, we proposed reconstructing a sensorimotor circuit for nociceptive temperature detection in a prosthetic hand to restore the amputees’ ability to warn and protect against noxious temperatures. Two tests were carried out to investigate how similar neuromorphic mechanisms implemented in prosthetic hand could allow amputees to release a hot object upon a nociceptive warning. Test 1 validated the feasibility of providing nociceptive warning and voluntary protection, while Test 2 was integrated with the neuromorphic withdrawal reflex strategy to provide nociceptive warning and protection in a reinforcing way. Results confirmed that integrating the hybrid sensory strategy with the neuromorphic withdrawal reflex control strategy created a voluntary or reinforcement protection effect. These strategies restored the amputees’ sensorimotor ability to warn and protect against nociceptive temperatures and enabled a prosthetic hand to provide a similar neuromorphic mechanism, which is in line with natural human motor control and proprioception.

The steady-state temperature prediction algorithm displayed robustness and accuracy in promptly detecting nociceptive temperature. This temperature prediction algorithm shortened the long response time of the thermosensitive sensor. Among the four different identification times for accuracy, 0.75 s was the value that balanced response speed and prediction accuracy. Compared to the long response time of 6 s, our identification time was significantly faster than the sensor’s own response time (Figure 2D). This prediction algorithm based on the sensor output characteristics could be applied to other sensors with the same long response time (Figure 2B).

The evoked hybrid sensation modalities could be perceived by switching the stimulation mode from the buzz range to the tingling range. Previous studies demonstrated that both buzz and tingling sensations could be perceived and discriminated clearly (Zhang et al., 2022b). In this study, we encoded the force and nociceptive temperature warning corresponding to buzz and tingling sensations. Both subjects achieved high perception success rates for force and nociceptive warning (Figure 5E). This hybrid sensory strategy was a further application for previous studies in ETS-based sensory feedback (Hao et al., 2020). In particular, the nociceptive warning perception performance of 94.3% was similar compared to those reported in the literature that involved temperature perception (range from 15°C to 40°C) at 97.2% (Iberite et al., 2023) or 88% (Ueda and Ishii, 2016). Based on the high perception performance, we found that there was no significant difference between the average PSE of subjects and the actual temperature warning threshold (*p* > 0.5, Figure 4A,B), which provided a feed-forward input for the adjustment of subsequent motion commands. Force perception was also important in regulating grip events. In a safe condition that the predicted temperature was below the warning threshold, subjects could maintain their grip force for 5 s with the linear buzz sensation feedback. These results demonstrated the ability of subjects to interpret the multi-modality tactile information based on the hybrid sensory strategy. Besides, a nociceptive warning could also encode information about a sharp object, which was also an important cue for prosthesis protection (Osborn et al., 2018). It is very useful to code hybrid information including normal and bursty states. Such a hybrid sensory coding strategy enriched the resolution of sensations and allowed subjects to interpret tactile information without heavy cognitive load.

The human reaction under nociceptive perception provides an excellent template for prosthetic hands to learn from. Inspired by the natural withdrawal reflex, a biomimetic withdrawal reflex for noxious temperatures was designed to generate a pair of reflex compensations to antagonistic muscles. The outputs of interneuron and synapse models manifested the biologically plausible spiking characteristics in the spinal cord as shown in Figure 3. The evoking of the withdrawal reflex was a consequence of the excitability and inhibition of the spinal reflex and descending to alpha motor commands. The increment in extensor muscle force and decrement in flexor muscle force corresponded to the reflex compensation that was superimposed on a pair of alpha motor commands. Figure 4 depicted the feasibility of adjusting reflexive alpha motor commands upon detection of nociceptive temperature. Results revealed that these alpha motor command adjustments could generate well-behaved muscle forces, which were consistent with the previous research (Niu et al., 2021; Zhang Z. et al., 2022). This withdrawal reflex originated in the spinal cord and was a demonstration of the short-latency spinal reflex, which was a subconscious action to unpredictable perturbation (Andersen, 2007; Zangrandi et al., 2021). This neuromorphic short-latency spinal reflex strategy could be also applied to other subconscious protective actions, such as grip force compensation and regulation upon detecting slippage (Osborn et al., 2016).

Since it was difficult to recognize potential heat hazards with the naked eye (Figures 5D,E), perceiving force and nociceptive temperature and adjusting motor commands based on current sensory feedback are valuable abilities of the human sensorimotor control system (Andersen, 2007; Moayedi and Davis, 2013). The withdrawal reflex and voluntary movement, whether synergistic or opposing, represent reinforcement and contradictory control modes. Both demonstrate the subject’s sensorimotor ability to nociceptive temperature, which are crucial for daily life activities. In this study, we focused only on the reinforcement mode as a proof of concept to verify the proposed sensorimotor strategies for prosthetic hands. Test 1 implemented and tested the voluntary release and Test 2 combined the neuromorphic withdrawal reflex as a reinforcement mode. Results in Test 1 proved that amputees restored this valuable ability to perceive force and nociceptive warning, allowing them to volitionally control the prosthetic hand after perceiving a tingling sensation (Figure 6). Figure 7 verified that the withdrawal reflex control strategy could tune motor commands and accelerate voluntary release, demonstrating the feasibility of the reflexive protection mechanism. There was no significant difference between each subject in all time indices despite amputees lack of various receptors. However, the AT, ET, and RT in Test 2 showed a significant difference from Test 1, and the PT was not significantly different from Test 1 (Figure 8), which confirmed that subjects were able to reinforce the protective effect in response to heat hazards.

There are still the following limitations. First, a constant threshold of 60.0°C was used for noxious temperature warnings. Considering that amputees mainly rely on the interaction with the contralateral heath hand in their daily activities, this study has verified the feasibility of mimicking the human hand’s nociceptive perception and withdrawal mechanism in a prosthetic hand. The nociceptive threshold temperature could be appropriately set to a critical value. This critical value in our approach could also be adjusted to avoid other potential damage using other sensors or prosthetic hands if needed. Second, the nociceptive temperature warning and protection study was tested on only two amputee subjects. The performance trends of both subjects were consistent and demonstrated that the neurocognitive ability of amputees was capable of processing two modalities of sensory information effectively. In future studies, this approach will be evaluated in more forearm amputee subjects to obtain more valuable data.

This study investigated and demonstrated the sensorimotor ability of amputees to perceive and protect from nociceptive temperature based on hybrid sensory feedback and neuromorphic withdrawal reflex strategies. Since this work was a proof-of-concept study, it focused on a temperature warning system that may provide protection for modern prosthetic hands from thermal damage. Furthermore, a nociceptor with learning capabilities will be designed to judge whether the current temperature is dangerous or safe based on synaptic plasticity (Wang et al., 2023). Future utilization of model-based software and neuromorphic hardware computation methods may facilitate the inclusion of additional neurons or even neural networks, enabling concise judgment of temperature warning threshold and precise modulation of withdrawal reflex. A linear sensory coding strategy to indicate pain intensity will be designed in the future (Coghill et al., 1999; Moayedi and Davis, 2013). Subsequent considerations will also include using the subject’s sEMG as an additional input to reflex model to modulate the reflex intensity. A more sophisticated control scheme will be employed to manage more complex scenarios in activities of daily life.

## 5 Conclusion

In this study, nociceptive temperature was detected swiftly by a steady-state temperature prediction algorithm with good robustness and accuracy, which compensated for the slow response time and improved the precision in the detection of nociceptive temperature. A psychophysical test showed that the proposed hybrid sensory strategy could restore the sensory ability of amputees to perceive force under safe conditions and nociceptive warnings when the temperature of grasped objects exceeded the warning threshold. Amputees were able to warn and protect against nociceptive temperature through voluntary release or reinforced by neuromorphic withdrawal reflex verified the feasibility of reconstructing the sensorimotor circuits for nociception and protection in prosthetic hands. This study confirmed that this prosthetic hand was capable of recapturing the hierarchical properties of heat warning and protection characteristic of the human hand, which was conducive to restoring the amputee’s ability to warn and protect against thermal nociceptive stimulus, and also provided embodiment and safety for amputees. More control schemes including reflex and voluntary intention in heat protection will be discussed in the future.

## Data availability statement

The original contributions presented in the study are included in the article/Supplementary material, further inquiries can be directed to the corresponding author.

## Ethics statement

The studies involving humans were approved by Ethical Committee of Human and Animal Studies at Shanghai Jiao Tong University (E2020021I). The studies were conducted in accordance with the local legislation and institutional requirements. The participants provided their written informed consent to participate in this study.

## Author contributions

AX: Data curation, Formal analysis, Software, Validation, Writing – original draft. CL: Data curation, Software, Writing – original draft. C-hC: Data curation, Software, Writing – original draft. TL: Resources, Writing – review & editing, Formal analysis. CD: Conceptualization, Supervision, Writing – review & editing, Project administration. NL: Funding acquisition, Project administration, Supervision, Writing – review & editing, Conceptualization.
