# Supplementary material for: A hybrid sensory feedback system for thermal nociceptive warning and protection in prosthetic hand
Source: Front Neurosci. 2024 Apr 8;18:1351348. doi: 10.3389/fnins.2024.1351348 (PMC11033464; doi:10.3389/fnins.2024.1351348)
Supplement: Supplementary file 1 [file Table_1.docx]

TABLE S1 Stimulation parameters of all subjects for the buzz and tingling sensory modalities.

| Subject | Age | Gender | Sensory  Modality | Amplitude  (mA) | Pulse Width  (μs) | Frequency  (Hz) |
| --- | --- | --- | --- | --- | --- | --- |
| A1 | 64 | Male | Tingling | 65 | 310 | 50 |
|  |  |  | Buzz (Index) | 55 | 210-270 | 50 |
|  |  |  | Buzz (Thumb) | 50 | 200-300 | 50 |
| A1 | 62 | Male | Tingling | 67 | 280 | 50 |
|  |  |  | Buzz (Index) | 50 | 180-280 | 50 |
|  |  |  | Buzz (Thumb) | 57 | 200-250 | 50 |
| H1 | 26 | Female | Tingling | 14 | 300 | 50 |
|  |  |  | Buzz (Index) | 12 | 180-280 | 50 |
|  |  |  | Buzz (Thumb) | 10 | 180-340 | 50 |
| H2 | 27 | Female | Tingling | 17 | 300 | 50 |
|  |  |  | Buzz (Index) | 10 | 180-300 | 50 |
|  |  |  | Buzz (Thumb) | 13 | 140-200 | 50 |
| H3 | 25 | Male | Tingling | 16 | 300 | 50 |
|  |  |  | Buzz (Index) | 12 | 180-250 | 50 |
|  |  |  | Buzz (Thumb) | 12 | 120-200 | 50 |
| H4 | 28 | Male | Tingling | 15 | 350 | 50 |
|  |  |  | Buzz (Index) | 10 | 150-250 | 50 |
|  |  |  | Buzz (Thumb) | 13 | 200-300 | 50 |
